# Exploring the accuracy of musical tempo memory: The effects of reproduction method, reference tempo, and musical expertise

**DOI:** 10.3758/s13421-024-01543-6

**Published:** 2024-03-20

**Authors:** Julia Vigl, Friederike Koehler, Heike Henning

**Affiliations:** 1 Department of Psychology, University of Innsbruck, Universitätsstraße 15, 6020 Innsbruck, Austria; 2 Department of Music Pedagogy, University Mozarteum Salzburg, Innsbruck, Austria; 3 Centre of Excellence in Music, Mind, Body and Brain, University of Jyväskylä, Jyväskylä, Finland; 4 Department of Music, Art and Culture Studies, University of Jyväskylä, Jyväskylä, Finland

**Keywords:** Musical tempo memory, Musical expertise, Tempo perception, Tempo reproduction, Tapping

## Abstract

Although people commonly remember and recreate the tempo of musical pieces with high accuracy, comparatively less is known regarding sources of potential variation in musical tempo memory. This study therefore aimed to investigate musical tempo memory accuracy and the effects of reference tempo, reproduction method, musical expertise, and their interaction. A sample of 403 individuals with varying levels of musical training participated in the experimental online study, including nonmusicians, amateur musicians, and professional musicians. Participants were tasked with reproducing the tempos of 19 popular pop/rock songs using two methods: tempo tapping and adjusting the tempo of the audio file based on the previously tapped tempo. Results from multilevel models revealed overall high accuracy in tempo memory, with tempo adjusting yielding greater accuracy compared with tempo tapping. Higher musical expertise was associated with increased accuracy in tempo production. In addition, we observed a quadratic effect of reference tempo, with the greatest accuracy in tempo reproduction around 120 bpm. Gender, age, familiarity with the pieces, and accompaniment strategies were also associated with greater accuracy. These findings provide insights into the factors influencing musical tempo memory and have implications for understanding the cognitive processes involved in tempo perception and reproduction.

**Supplementary Information:**

The online version contains supplementary material available at 10.3758/s13421-024-01543-6.

As a fundamental element of music, tempo affects how we respond to music emotionally and physically (e.g., through conveying different moods or expressive intentions). For instance, faster tempos are usually associated with energy or happiness while slower tempos relate to relaxation or sadness (Liu et al., 2018). In general, tempo reflects the speed of a musical piece commonly measured by regular beats per minute (bpm), while beats are often characterized as the quasiperiodic pattern of salient points in time (Nave-Blodgett et al., 2021). While in modern musical scores the desired number of bpm is usually indicated by a metronome mark, musical notation can also include tempo indicators, such as *Andante* or *Allegro*, allowing for a range of bpm. Beyond the mere understanding of tempo as a series of isochronous time units, tempo also helps to connect successive musical events in a piece contributing to the whole musical experience (Gratton et al., 2016). How we remember and recreate musical tempo therefore is crucial for musical expression and appreciation (Foster et al., 2021), not only for musicians but also for music listeners. For instance, people listening to music naturally feel the urge to rhythmically move their bodies to the beat (Senn et al., 2019), which is why finger tapping to an external or imaginary rhythm has emerged as a popular tool for research on tempo perception and production.

## Musical tempo memory

The ability to remember and reproduce the tempo of musical pieces is often termed musical tempo memory (e.g., Gratton et al., 2016; Rashotte & Wedell, 2014). Musical tempo memory appears to emerge quite early in young infants’ development (Trainor et al., 2004). However, although human brains are generally structured to process musical information, musical tempo memory consists of a complex interplay between auditory and motor processes activating similar brain regions, such as basal ganglia and sensorimotor cortex (Hayes et al., 2020). Over the past decades, extensive research in music cognition has confirmed the existence of a long-term memory for musical information (e.g., Levitin, 1999; Trainor et al., 2004), while some literature also points to an additional working memory faculty containing only musical information (Berz, 1995). In particular, studies have shown that specific musical features, such as tempo or pitch, are stored comprehensively in music listeners even without considerable musical training (e.g., Levitin & Cook, 1996). Despite a lack of research on the processes underlying musical memory, it has been suggested that such auditory features of music may be stored in a memory system encoding both sensory and abstract information from the music to form musical representations (Peretz & Zatorre, 2005). While sensory information includes the mere perceptual qualities of music, such as loudness or pitch, abstract musical information allows storage and recognition of music despite changes in those perceptual aspects (e.g., when the melody is transposed to a different register, or when the instrumentation is changed). Naturally, musical tempo memory is therefore related to general human time perception and temporal memory (i.e., the ability to remember and recreate the length of any time units; Grondin, 2005) but distinct through a neural embedding as part of the whole musical experience with sensory and auditory features. The investigation of musical tempo memory thus can reveal how the human brain processes and stores mental representations of musical information providing insights into general cognitive processes and abilities of human beings.

In previous research, individuals were able to remember and recreate the original tempo of a musical piece with a remarkably high accuracy. In an early demonstration, Levitin and Cook (1996) recorded participants singing their favourite songs and found that 72% of the recordings were within ±8% of the original tempo. Similarly, research demonstrated high accuracy of tempo discrimination between original and tempo-altered versions of familiar songs (Hayes et al., 2020). Even the tempo of unfamiliar music, that is, melodies heard only twice, can be remembered well (Schellenberg et al., 2014). Further, research on involuntary musical imagery (or “earworms”), which involves the spontaneous recall and mental replay of a tune, song, or musical piece, provides evidence for highly accurate tempo recall from long-term memory in everyday life (Jakubowski, Farrugia et al., 2015a). Correspondingly, temporal accuracy was similar between involuntary and deliberate recall of songs (Jakubowski et al., 2018). In addition, musical tempo memory appears to be quite stable over time. For instance, mothers were recorded singing to their infants in nearly identical tempos over two measurement occasions (Bergeson & Trehub, 2002), while musicians’ recordings of exclusively orally transmitted Aboriginal songs deviated in tempo only marginally over a long time period (Bailes & Barwick, 2011).

## Contextual and individual factors in musical tempo memory

While these studies provide evidence for a generally high accuracy and consistency of musical tempo memory, comparatively less is known about contextual and individual factors influencing the accuracy of recalling musical tempo. For instance, previous research indicates that representations of tempo might vary more than other musical features, such as pitch information (Janata & Paroo, 2006). Indeed, studies focusing on tempo memory through comparing original songs and tempo-altered versions provide evidence for differential context effects (Hayes et al., 2020; Rashotte & Wedell, 2014).

One contextual factor influencing musical tempo memory might be the reproduction method with which musical tempo is recalled and recreated. For example, a recent study (Jakubowski et al., 2016) compared a perceptual music task (with all musical cues present) with a task engaging the motor system (tapping in time to the imagined music) and a nonmotor task (adjusting a click track to the imagined music). Indeed, analyses revealed that tempo accuracy in familiar songs was highest in the perceptual music task, while the motor task yielded better performance than the nonmotor task. Corresponding to research on pitch memory (Janata, 2012), the greater sensory support for tempo recall during the perceptual music task might have enhanced accuracy. However, the sample size of the study by Jakubowski et al. (2016) was rather small (*N* = 25) and mostly female which requires replication and further investigation of their findings with a larger and more diverse sample.

Another source for variation in accuracy of musical tempo memory might be the reference tempo, that is, the original tempo of a song. For example, Jakubowski et al. (2016) found that participants reproduced songs with a faster original tempo with higher accuracy than slower songs. However, in this study, the fastest original tempo was around 120 bpm, which overlaps with the generally preferred perceptual tempo of adults of 100–120 bpm (McKinney & Moelants, 2006). Therefore, more research based on a wider range of tempos is necessary to investigate how musical tempo memory might vary with different tempo.

Furthermore, individual factors may also play a role in musical tempo memory, based on the high variability in accuracy between individuals (Jakubowski et al., 2016). In particular musical expertise might significantly account for individual differences since musicians generally show better temporal discrimination and rhythm detection (Rammsayer & Altenmüller, 2006) as well as better detection of tempo changes (Sheldon, 1994) compared with nonmusicians. One explanation might be an enhanced auditory-motor integration of neural systems in the context of musical rhythm in musicians (Chen et al., 2008). Moreover, musical training has been associated with increased mental imagery ability, especially in the auditory domain (Aleman et al., 2000). This proficiency in mentally simulating music might explain why musicians in Jakubowski et al. (2016) showed particular advantages when reproducing the tempo of an imagined piece through tapping, while no significant differences were found between the groups in other conditions. However, most of these studies merely compared musicians with nonmusicians overlooking the group of amateur musicians who might differ in their musical tempo memory abilities. Despite the evidence on positive effects of musical expertise, other studies’ findings are inconsistent. For instance, Fine and Bull (2009) instructed musicians and nonmusicians to recreate three specific tempi from memory by clapping, but found no significant differences. In addition, another study showed that musical training explained differences in tempo identification but not in production tasks (Gratton et al., 2016). However, most of the studies on the effects of musical expertise employed unfamiliar or artificially created music highlighting a particular lack of research on musical tempo memory regarding familiar songs. Further, small sample sizes might have impaired interpretability and generalizability of these findings.

## The present study

The present study investigated musical tempo memory based on familiar songs without external reference to address the gaps and inconsistencies in previous research. Specifically, the aim was to examine individuals’ accuracy of musical tempo memory regarding the influence of three potential sources of variation: reproduction method, reference tempo, and musical expertise. The present study thus extends past research in terms of higher sample size and a more representative sample, manipulation of reproduction method (imagined music task, i.e., tapping of tempo, versus perceived music task, i.e., adjusting of tempo), reference tempo (including a wider bpm range in songs), and a continuous assessment of musical expertise, overcoming the dichotomy of nonmusicians versus professional musicians.

Based on the literature, we formulated the following hypotheses:

- *H1:* There is a positive association between the tempo of pieces and accuracy: Faster tempos are reproduced more accurately than slower pieces.
- *H2:* Accuracy of tempos is higher when tempo is adjusted than when tempos are tapped without external reference.
- *H3:* The higher musical expertise, the better individuals are able to reproduce tempos of familiar musical pieces, both in tapping tempo and in adjusting the tempo of the audio files.
- *H4:* There is an interaction between reproduction method and musical expertise. The difference in accuracy between individuals with low and high musical expertise is greater when tapping the tempo than when adjusting the tempo of the audio file. In addition, we conducted an exploratory analysis to examine whether other interactions exist between musical expertise and predictors or control variables.

## Method

### Participants

A total of 431 individuals participated in the study. After excluding two participants with a very high average tapping rate above 400 bpm and 26 participants with very irregular tapping patterns (average standard deviation of taps greater than 20% of the average tapping rate), the final sample comprised 403 participants (59% female, 40.7% male, 0.5% nonbinary). They had an average age of 26.91 years (*SD* = 10.38, range: 16–69) and mostly lived in Austria (65%), Italy (17%), or Germany (16%). The majority of participants held a high school diploma (47%), followed by a university degree (35%), vocational higher education (10%), or compulsory education (8%) as their highest educational qualification.

With regard to self-reported musician status, 26% (*n* = 105) of participants considered themselves nonmusicians, 34% (*n* = 137) were amateur musicians, and 40% (*n* = 161) were semiprofessional or professional musicians. As shown in Table S1 of the Supplementary Materials, there were no significant differences among these groups in terms of demographics, weekly music listening, or familiarity of the songs. Amateur musicians liked the songs slightly more than the other two groups. As expected, significant and strong differences were observed in music-related variables, such as playing an instrument or having choral experience, length of time playing an instrument, and hours of weekly practice, with a higher self-reported musical status being associated with more experience and practice.

### Procedure

The study was conducted online using the LimeSurvey software (Version 2.64; Limesurvey GmbH, 2022), and participants were recruited through email invitations sent to all students by both the University and Mozarteum conservatory of Innsbruck in Austria. Additionally, participants from various psychology and music education courses were encouraged to motivate friends and acquaintances to participate in the study. As an incentive to participate, subjects received a graphically presented overview of the achieved accuracy scores per song and in total at the end of the study. In accordance with the protocol of the Mozarteum conservatory, ethical approval was not deemed necessary for this study. The research project did not involve the collection of sensitive data, and participation in the study was voluntary, ensuring complete anonymity for all participants.

**Fig. 1:**
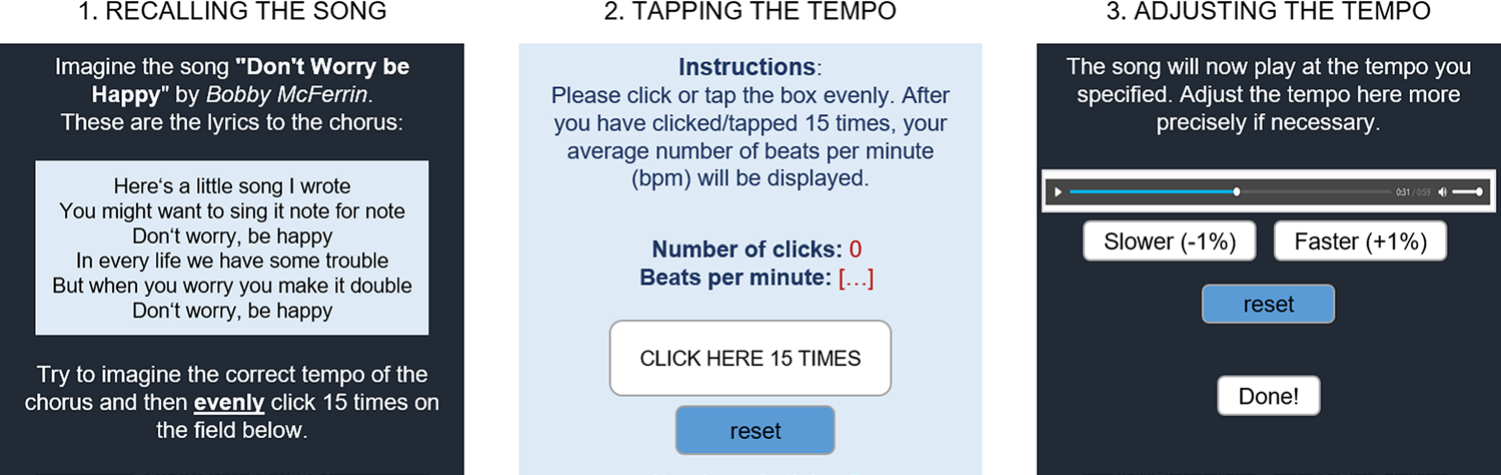
Visualization of the three steps of tempo reproduction

After the subjects gave their informed consent to participate, they were asked to select those songs from a list of 18 with which they were familiar enough to mentally sing along with the melody of the chorus. The lyrics of the chorus were displayed as a prompt for each song. Participants could only proceed with the study if they selected at least half of the songs (nine songs). Subsequently, they were asked questions about their mood, demographic aspects, and musical experiences. Then, participants were introduced to the two methods for measuring tempo memory and underwent a familiarization task: First, they had to tap evenly on our application (see section Assessment of Musical Tempo) and reproduce the rhythm of a clock’s second hand. Then, an audio file of a ticking clock was played at the tempo tapped by the participant and the speed of the audio file could be adjusted. Participants received feedback on their accuracy and were instructed to do the same for the chosen songs. It was emphasized that the tempo of the tracks should be tapped evenly (as the second hand does) and that no rhythmic patterns should be tapped. Participants subsequently provided their musical tempos for all selected songs in random order using both methods, and reported how familiar and enjoyable each song was to them. Figure 1 illustrates the three stages of tempo reproduction, which include (1) recalling the song from memory without listening to it, (2) tapping the tempo, and (3) adjusting the playback speed of the audio file played at the tempo provided in Step 1.

### Materials

#### Songs

The pop/rock songs used were selected in a preliminary study. We gathered a list of 80 songs with a wide range of different bpms (range: 52–184) that are very well-known, for example, due to their presence on websites with the most popular songs, being in charts, or their large Spotify listenership. In an online survey using the SosciSurvey software (Leiner, 2019), 69 participants (81.2% female, mean age = 33.57 years, *SD* = 17.54) were presented with this list of songs and asked to select the songs that were familiar enough that they could spontaneously recall the melody and lyrics of the chorus. Eighteen pieces were selected from the initial list based on their high level of familiarity. The aim was to include well-known pieces for all three age categories identified in the preliminary study by tertile split (<23 years, 23–40 years, over 40 years). Overall, for each age group, at least 10 of the 18 pieces were known by at least 60% of the participants. Their bpm ranges from 53 to 169 and all songs are written in 4/4 time signature. Table S2 provides a complete list of the 18 selected songs, their bpm and their familiarity percentages.

#### Assessment of musical tempo

We assessed the recalled tempo of the selected music tracks using two methods. First, participants evenly tapped the tempo from memory (“tapping tempo”). Second, they adjusted the playback speed of the audio file starting from the speed of their tapping (“adjusting tempo”).

Regarding *tapping tempo*, we employed a slightly modified version of the application developed by Vigl et al. (2023), which was written in JavaScript using the JQuery library (Version 3.4.1). Participants were asked to click (if on a laptop/PC) or tap (if on a smartphone) evenly 15 times at the tempo of the piece. The application calculates the tempo of the clicks in bpm by measuring the time intervals between each click. We adapted the application to extract additional values, namely not only average bpm of clicks after 15 taps, but further the list of 15 bpms in order to examine tapping stability, which were stored in the survey’s variables.

Regarding *adjusting tempo*, participants adjusted the playback speed of the song audio file starting from the speed of their tapping. Throughout this process, the pitch and frequency of the music remained unchanged, regardless of whether the tempo was slowed down or accelerated. To ensure that the audio file would play correctly, we cleaned up the metre using a specially generated function (see clean_bpm() in the Supplementary Material): If someone tapped faster than 150% of the target tempo, the bpm number would be halved until the bpm was within 150% of the target tempo. If someone tapped slower than 75% of the target tempo, the bpm number would accordingly be doubled once or more times.^1^ This was done such that the audio file would be played correctly even if someone has tapped in half or double time, for example, tapping the tempo of eighth notes instead of quarter notes. Participants could then adjust the playback tempo by ±1% of the tapped start tempo using two fields below the audio player. Finally, participants submitted the adjusted tempo.

The functions used for assessing musical tempo by tapping and adjusting are available in the Supplementary Material, section *Code for Assessment of Musical Tempo*.

#### Musical tempo accuracy score

To assess accuracy, we calculated an unsigned accuracy score of tempo memory using the formula of Vigl et al. (2023) presented in Eq. 1. In contrast to accuracy scores of previous studies, this formula considers the logarithmic nature and sinusoidal fluctuations of the bpm scale, which means that it accounts for the fact that the distances from the correct tempo are not linear, but logarithmic, and double or half tapped tempos can also correspond to an accurate tempo. For instance, if the correct tempo is 100 bpm in quarter notes, tapping the eighth notes at 200 bpm or the half notes at 50 bpm would be equally correct. The formula produces an accuracy score between 0 and 1, with higher scores indicating higher accuracy.

*Score of unsigned tempo accuracy from *Vigl et al. (2023)*. The term bpm refers to the tempo given by the participant; the term ref refers to the correct reference tempo.*

1 $$(Cos(Log(bpm/ref)*\mathrm{20,87})+1)/ 2$$

Figure S1 illustrates which unsigned scores would be obtained by a participant’s response at different tempos, using an example reference tempo of 100 bpm. In the main analysis (i.e., the multilevel models), the unsigned scores per songs were included, while for the correlation matrix, an average was taken of the accuracies per person across all selected songs. In addition, from the list of individual taps for each song, the stability of tapping was calculated at the relative standard deviation (standard deviation / mean tapping tempo) and averaged over all rated songs for the correlation matrix. For tapping stability, lower values thus indicate higher stability.

#### Questionnaires

##### Musical expertise

We employed a set of questions to inquire about participants’ musical experiences. Firstly, we asked them to self-assess their musical status (1 = *nonmusician*, 2 = *music-loving nonmusician*, 3 = *amateur musician*, 4 = *semiprofessional musician*, 5 = *professional musician*). Additionally, we asked whether participants had any experience playing an instrument or singing, or participating in a choir (0 = *no*, 1 = *yes*), the number of years of instrumental practice they had, the number of hours per week they spent practicing their instrument or singing (0 = *no practice*, 9 = *20 hours and more*), and how often they listen to music weekly (1 = *never*, 5 = *every day*). These questions were *z* transformed and combined into a continuous composite score of musical expertise (α = .71), with the exception of the music listening question. The composite score has a mean of 0 due to the previous standardization, with higher scores indicating greater musical expertise. In the correlation matrix and multilevel models, we used the composite score as the measure of musical expertise. When comparing the groups in Table S1 and for visualization purposes in the figures, we used a recoded version of the variable assessing musical status (1 = *nonmusician*, 2 = *amateur musician*, 3 = *semiprofessional or professional musician*), as this variable was highly correlated with musical expertise (*r* = .83, *p* < .001).

##### Mood and alertness

Since mood and arousal showed significant associations with both general time perception (e.g., Droit-Volet, 2013), as well as musical tempo memory (e.g., Jakubowski, Halpern, et al., 2015b) in previous research, we included mood and alertness as covariates. In the present study, we assessed positive and negative mood at the start of the survey with the I-PANAS-SF (Thompson, 2007), with five items for positive affectivity (α = .71) and five items for negative affectivity (α = .65), being rated on a 5-point scale (1 = *not at all*, 5 = *very much*). In addition, we asked how awake participants felt (0 = *very tired*, 10 = *very awake*).

##### Music-related variables

To account for individual differences in music-related variables, we measured the use of accompanying strategies as well as personal familiarity and liking of the song for inclusion as covariates. At the end of the survey, we asked participants whether and how often they used four accompaniment strategies (singing internally, moving both hands, tapping their toes, moving in time with the music) while clicking/tapping musical tempos (1 = *never*, 5 = *for all songs*). For the analyses, we computed a mean value from these answers. Further, participants rated their familiarity with and liking of all songs on a 5-point scale (1 = *not at all*, 5 = *very*).

### Data analysis and power

All four hypotheses were analyzed using multilevel models. This approach allowed us to examine the joint effects of tempo, method, musical expertise, and other participant variables, while accounting for repeated measures within participants due to multiple songs and two reproduction methods (tapping tempo vs. adjusting tempo). To investigate if a multilevel approach is suitable and to determine the best fitting model, we first built models step by step and compared the Bayesian information criterion (BIC). Preference was given to models with lower BIC values due to their better fit to the observed data and their ability to provide a balance between model simplicity and accuracy. We evaluated models with random intercept, random slope of tempo, and random slope of tempo and method. The model with random intercept and random slope of tempo provided the best fit and was therefore selected as the initial model (see Table S3), while the model with random slope of tempo and method failed to converge and was thus not used. To test Hypothesis 1 regarding the relationship between reference tempo and accuracy, we included both the reference tempo and its quadratic term as predictors in the analysis. This approach allowed us to determine whether the model fit improved with the inclusion of the quadratic term, thereby investigating the presence of a linear or curvilinear effect of tempo. For Hypotheses 2–4, we included reproduction method and musical expertise as predictors, as well as the interaction between method and musical expertise. We also included several control variables in the model, namely age, gender, mood, tapping stability, accompanying strategies, familiarity and liking of the corresponding song. To enable a direct comparison of coefficients, we standardized all but binary predictors to have a mean of zero and a variance of one before computing the models.

Statistical power for detecting main and interaction effects in multilevel models is influenced by various factors such as effect sizes, standard deviation of slopes at lower levels, sample size at both levels, and ICC. As some of these variables were not known, it was not possible to calculate an optimal sample size using a priori power analysis. However, based on previous literature recommending a minimum sample size of 50 clusters at the highest level (McNeish et al., 2017) and the sample size requirements for a linear regression with 16 predictors, which requires at least 204 subjects to achieve 95% power with a mean effect size (*f*^2^ = 0.15) according to G*Power (Faul et al., 2009), we concluded that the current sample size of 403 participants would be sufficient.

## Results

### General results

**Fig. 2:**
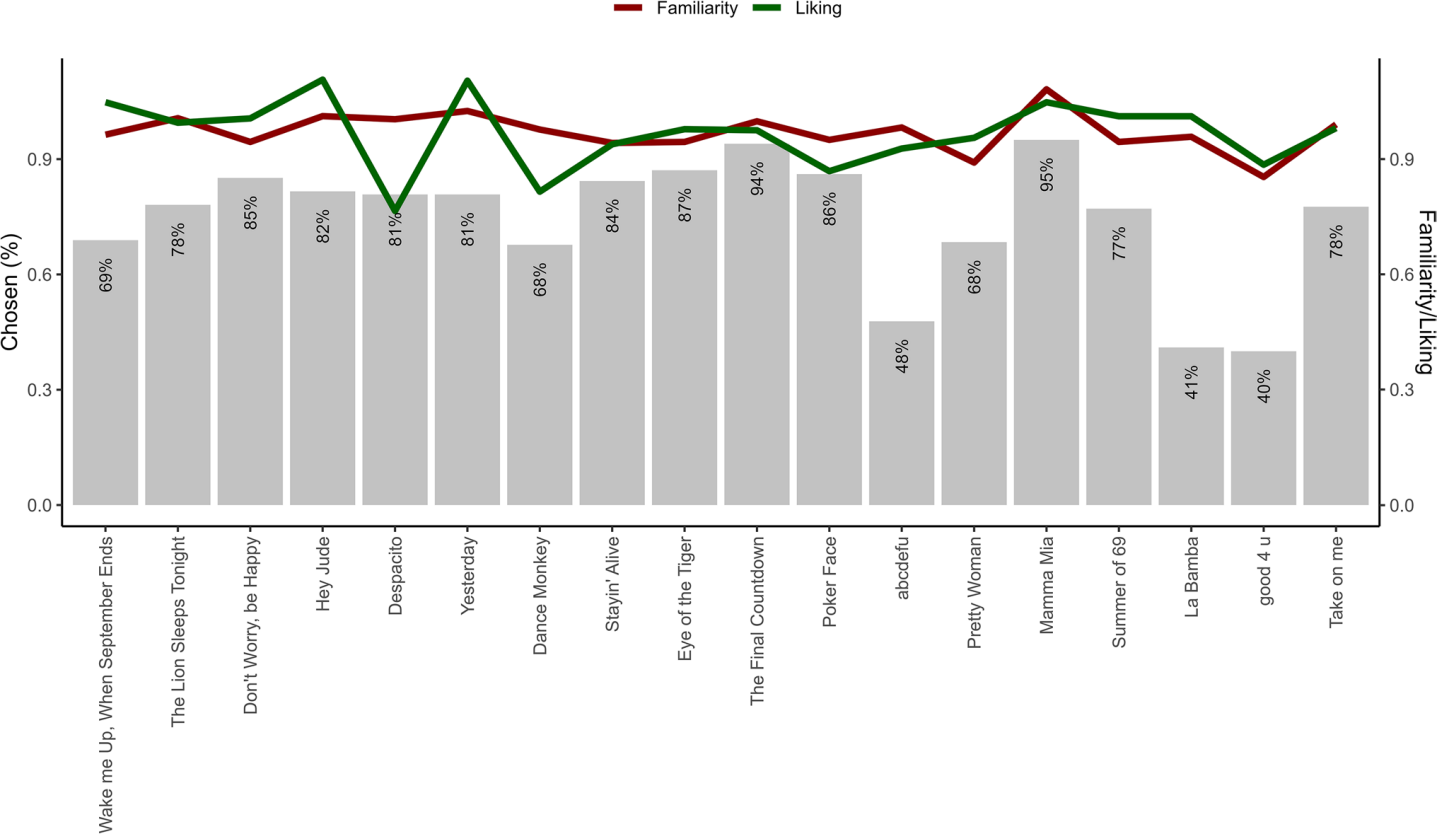
Percentage of participants who selected the songs (bars), as well as their familiarity and liking (lines). Songs are sorted according to their reference tempo

On average, participants selected 13.41 (*SD* = 2.76, range: 9–18) of the 18 songs, which were moderately familiar (*M* = 3.61, *SD* = 0.59) and liked (*M* = 3.47, *SD* = 0.58). Figure 2 shows the percentage of participants who chose each of the songs (bars) and how well-known and liked the same songs were to the participants choosing them (lines). For this illustration, familiarity and liking, which were actually measured on a scale of 1 to 5, were rescaled to values between 0 and 1. As can be seen there, most of the pieces were chosen frequently, with the exception of three songs, which were chosen only by a smaller group of individuals. Of note, the exclusion of these three songs did not alter our findings, which is why we included data on all songs in our main analyses.

Table 1 shows the descriptive statistics and correlations between assessed variables. As anticipated, adjusting tempo was associated with higher accuracy of musical tempo memory compared with tapping tempo.^2^ Overall, we found weak significant correlations between accuracy and musical expertise, accompanying strategies, and familiarity with the songs, which were slightly higher for tapping scores. Gender and age were negatively correlated with accuracy, suggesting that women and older participants tended to perform poorer. There were no associations between mood, alertness, and liking of the songs and accuracy of musical tempo memory. With higher musical expertise, participants used the accompanying strategies more frequently.

**Table 1:** Descriptive statistics and zero-order correlations

	*M* (*SD*)	1	2	3	4	5	6	7	8	9	10	11	12
1 Gender::Female^a^	–	–											
2 Age	26.91 (10.39)	−.16^**^	–										
3 Education	4.19 (1.21)	.05	.32^**^	–									
4 Positive Mood	3.13 (0.64)	.09	.03	−.09	–								
5 Negative Mood	1.27 (0.38)	−.01	−.09	.01	−.05	–							
6 Alertness	5.67 (2.18)	.01	.01	−.09	.01	−.05	–						
7 Musical Expertise	0.00 (0.85)	.06	.07	.14**	.01	−.01	−.10*	–					
8 Accompanying Strategies	3.54 (0.88)	.03	−.06	.06	.09	−.04	.04	.33^**^	–				
9 Familiarity	3.61 (0.59)	.13^**^	−.03	−.01	.28^**^	−.09	.11*	.07	.15^**^	–			
10 Liking	3.47 (0.58)	.14^**^	−.05	−.04	.27^**^	−.12^*^	.13*	.09	.22^**^	.64^**^	–		
11 Tapping Stability^b^	0.08 (0.03)	.10*	.08	−.09	.03	−.03	.10*	−.38**	−.40**	−.20**	.14**	–	
12 Score Tapping	0.76 (0.14)	−.14^**^	−.19^**^	−.01	.00	−.03	−.09	.23^**^	.18^**^	.14^**^	.08	−.41**	–
13 Score Adjusting	0.87 (0.09)	−.13^**^	−.15^**^	−.04	.01	−.04	−.04	.18^**^	.12^*^	.11^*^	.05	−.34**	.64^**^

^a^ Gender was coded as 0 = nonfemale (male or nonbinary), 1 = female. ^b^ Tapping Stability: Lower values indicate higher stability

* *p* < . 05. ** *p* < . 01

Participants seem to have had a good understanding of the task, as indicated by the high mean tapping stability (*M* = 0.08, *SD* = 0.04), which reflects the relative standard deviation of individual taps from the mean tempo. Stability was higher with higher musical expertise, higher familiarity with the songs, and more accompanying strategies. The stability in tapping (*M* = 0.08, *SD* = 0.11) and the accuracy in both tapping (*M* = 0.66, *SD* = 0.35) and adjusting (*M* = 0.72, *SD* = 0.32) the second hand during the familiarization task were similar. However, there was no difference between the three groups of musical expertise in terms of score and stability in the familiarization task.

The designated metre was tapped in 71.4% of all tapping instances, while 16.7% of the tapping was done at half the reference tempo and 11.8% at double the reference tempo. Figure S2 visualizes the percentage of participants tapping in designated, half, or double metres for each reference tempo. As evident there, participants mostly tapped in the expected metre; double metres were more common at slower tempos, and half metres at faster tempos.

### Musical tempo accuracy at different reference tempos

**Fig. 3:**
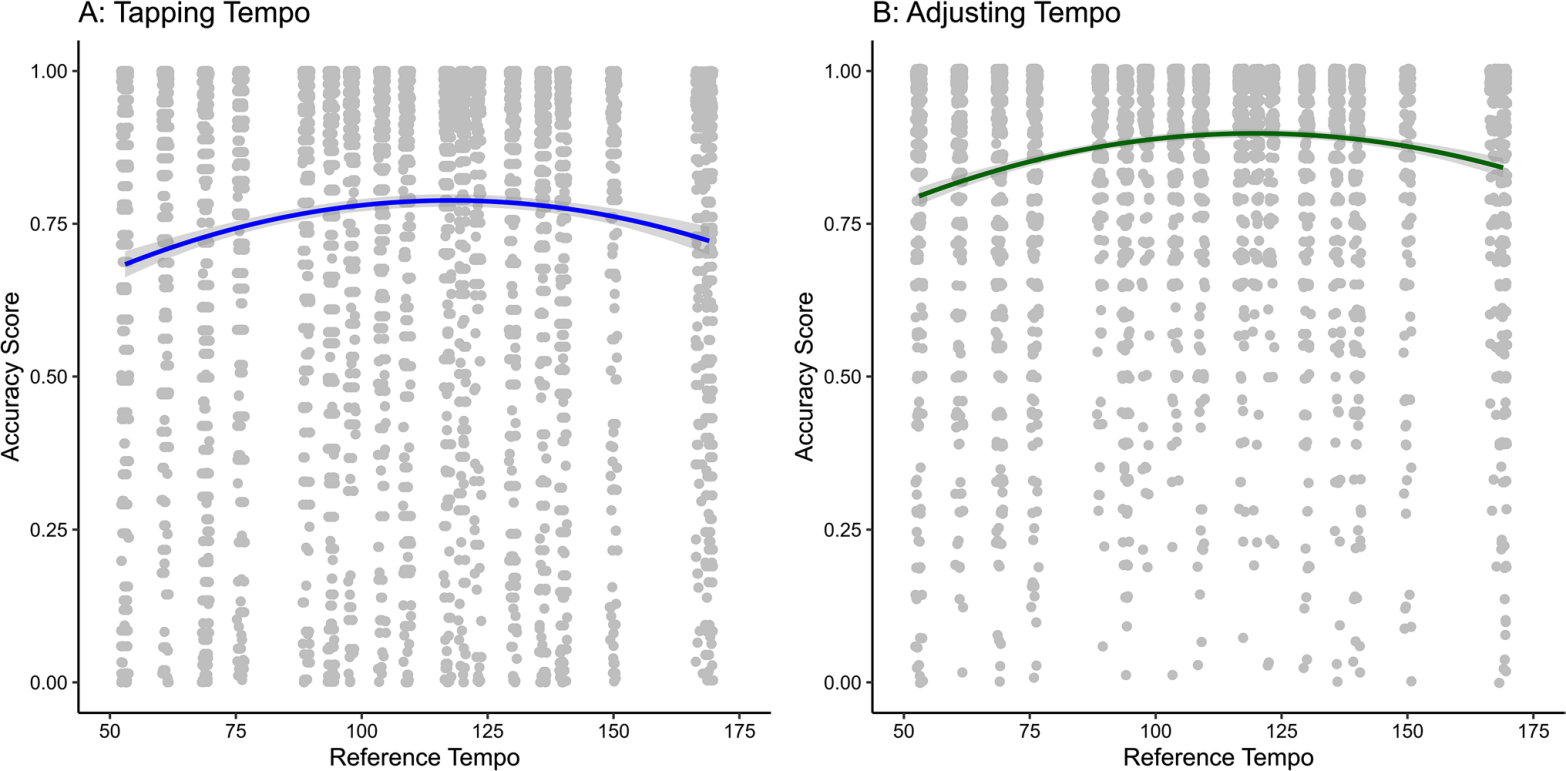
Quadratic tempo effect on the accuracy of musical tempo memory for tapping (**A**) and adjusting the audio file (**B**). *Note.* The dots represent participants’ individual accuracy scores for each song. The lines show the quadratic effect of song tempo on accuracy for both tapping and adjusting the tempo

To test Hypothesis 1, we fitted two multilevel models, one with a linear effect of tempo and the other with both linear and quadratic effects. As shown in Table S4, tempo had a significant linear effect (*r* = .20, *p* < .001) and that adding the quadratic effect explained additional variance (linear: *r* = .13, *p* < .001; quadratic: *r* = .12, *p* < .001). Model comparison using BIC revealed that the model with the quadratic effect provided a significantly better fit than the linear model, χ^2^(1) = 152.57, *p* < .001, BIC = −673.85 vs. −530.57. Figure 3 illustrates this quadratic relationship, where the highest accuracy is observed around a tempo of 120–125 bpm. This effect holds for both tapping tempo (Fig. 3A) and adjusting tempo (Fig. 3B), although the latter method generally yielded higher accuracy. Hypothesis 1 can therefore only be partially confirmed: there was an effect of the tempo of a piece of music on the accuracy, but it was a quadratic and not a linear effect.

### Predictors of accuracy

Hypotheses 2, 3, and 4 postulated an effect of method (tapping vs. adjusting tempo), an effect of musical expertise, and an interaction of both on musical tempo memory accuracy. Table 2 presents the results of the multilevel regression model with all predictors and control variables. Because gender and age showed significant correlations with musical tempo accuracy, we included them in our control variables. To enhance the interpretability of the coefficients, we present them additionally as Pearson’s *r* coefficients.

**Table 2:** Multilevel model predicting accuracy score

	Accuracy Score			
Predictors	Est.	CI	*p*	*r*
(Intercept)	0.89	0.88, 0.91	**<.001**	
* Tempo*	0.23	0.20, 0.26	**<.001**	.15
Tempo^2^	−0.20	−0.23, −0.18	**<.001**	−.14
Method::Tapping^a^	−0.11	−0.12, −0.10	**<.001**	−.26
Musical Expertise	0.01	0.00, 0.02	**.047**	.09
Method × Musical Expertise	0.02	0.01, 0.03	**.001**	.04
Control Variables				
Gender::Female^b^	−0.04	−0.06, −0.02	**<.001**	−.20
Age	−0.02	−0.03, −0.01	**<.001**	−.22
Positive Affectivity	0.00	−0.01, 0.02	.516	.03
Negative Affectivity	−0.00	−0.01, 0.00	.314	−.05
Alertness	−0.01	−0.02, 0.00	.109	−.08
Chosen Metre	0.03	0.03, 0.04	**<.001**	.11
Tapping Stability^c^	−0.01	−0.02, −0.01	**<.001**	−.05
Familiarity with Song	0.02	0.01, 0.02	**<.001**	.06
Liking of Song	−0.00	−0.01, 0.00	.146	−.01
Accompanying strategies	0.01	−0.00, 0.02	.191	.07
Random effects				
σ^2^	0.05			
τ_00 id_	0.01			
τ_11 id.Tempo_	0.00			
ρ_01 id_	0.08			
ICC	0.17			
N _id_	403			
Observations	10812			
Marginal *R*^2^/Conditional *R*^2^	0.112 / 0.264			

Effect-size *r* was calculated as √(*t*^2^/(*t*^2^ + *df*)) (Rosnow & Rosenthal, 2008)

CI = 95% confidence intervals; Est. = estimates of unstandardized coefficients;

σ^2^ = within-group variance, residual variance; τ_00 id_ = between-group variance, random intercept of participant (with random slope of tempo); τ_11 id.Tempo_ = variance of the random slope of tempo at participant level; ρ_01 id_ = correlation between random intercept of participant and random slope of tempo; ICC = intraclass correlation coefficient

^a^Method::Tapping was coded as 0 = adjusting tempo, 1 = tapping tempo

^b^Gender::Female was coded as 0 = male or nonbinary, 1 = female

^c^Tapping Stability: Lower values indicate higher stability

**Fig. 4:**
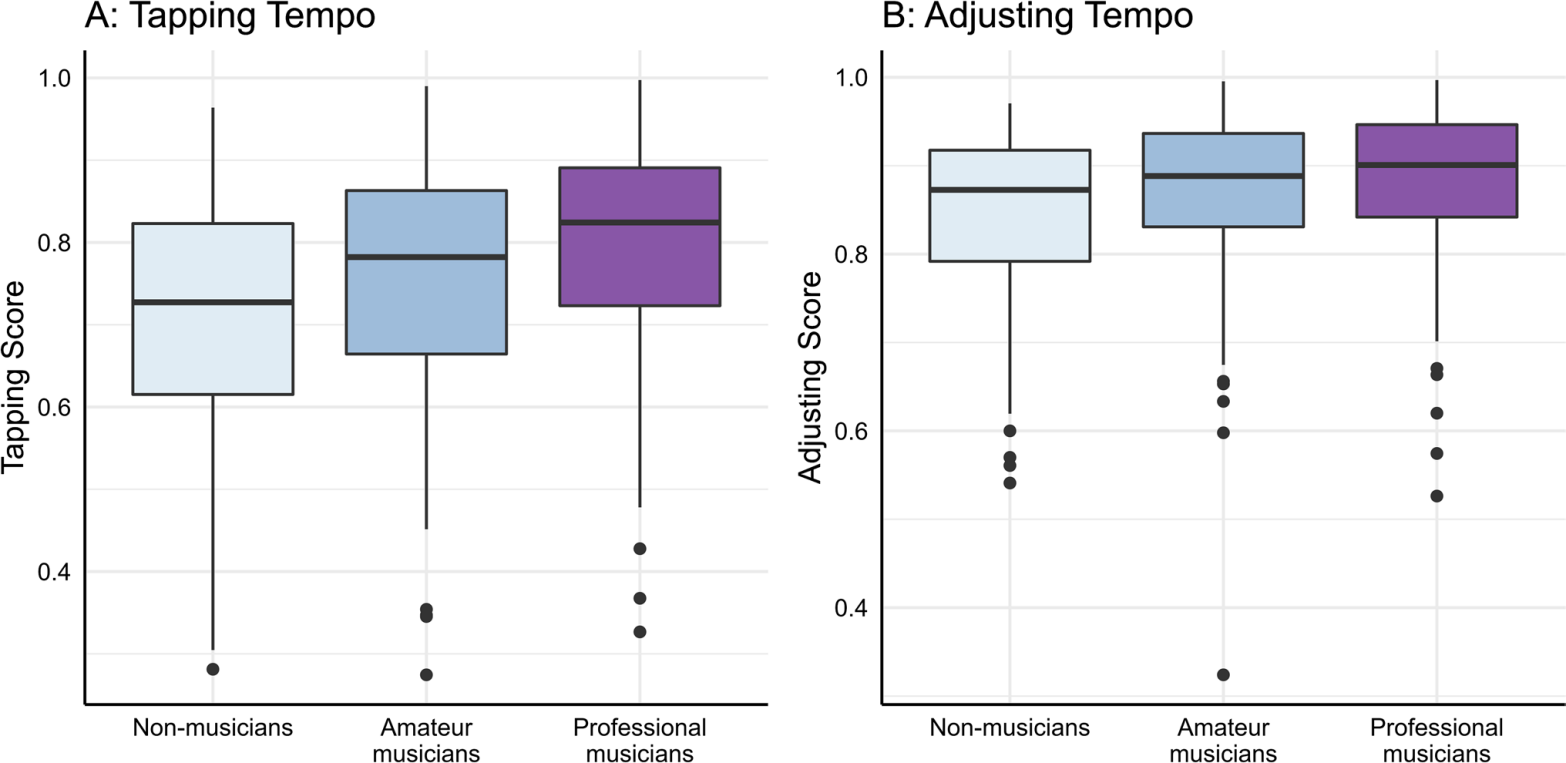
Differences between nonmusicians, amateur musicians, and professional musicians regarding accuracy when tapping the tempo (**A**) and adjusting the audio playback speed (**B**). *Note.* In the box plot, the box represents the interquartile range between the first and third quartiles, encompassing 50% of the data. The whiskers represent data outside the first and third quartiles. Individual points outside the whiskers represent possible outliers

The multilevel model revealed significant effects for all expected predictors, with the method having the strongest effect. This indicates that accuracy was lower when participants tapped the tempo compared with when they adjusted the audio track. Additionally, a significant linear and quadratic effect of tempo was observed again, along with a small main effect of musical expertise, where higher expertise was associated with higher accuracy. Finally, there was an interaction between method and musical expertise. As illustrated in Fig. 4, this small effect suggests that the effect of musical expertise is stronger in tapping compared with adjusting.

As previously noted in the correlation matrix, age and gender emerged as significant predictors among the control variables. The selected metre—half tempo, target tempo, or double tempo—also had a significant effect on accuracy, with faster tapping resulting in more precise performance. Higher tapping stability was associated with a higher accuracy. Familiarity with the song showed a weak effect, while liking and other control variables did not significantly impact accuracy. Accounting for these variables reinforces the stability of the expected effects even when other predictors are controlled for. Notably, the quadratic effect of reference tempo, the effect of musical expertise, and the interaction between expertise and method remain robust even when the tapping stability and the chosen metre are taken into account. Hypotheses 2,3, and 4 can thus be confirmed.^3^

## Discussion

The aim of the present study was to investigate how musical tempo memory accuracy is influenced by reference tempo, reproduction method, musical expertise, and the interaction between musical expertise and method. Using a large sample of over 400 individuals with varying degrees of musical expertise, ranging from nonmusicians to amateur and professional musicians, we found that overall tempo memory accuracy was remarkably high. However, tempo adjusting was associated with higher accuracy compared with tempo tapping, and individuals with higher musical expertise displayed enhanced accuracy. Notably, musically trained individuals had a specific advantage in tapping, as evidenced by the interaction between musical expertise and reproduction method. Furthermore, we observed a quadratic effect regarding reference tempo, indicating that tempos around 120 bpm were reproduced with greater precision compared with faster or slower tempos. In the subsequent sections, we will summarize our findings, establish connections to previous literature, and discuss the implications for both research and practical applications.

### Predictors of accuracy of musical tempo memory

In this study, we incorporated various factors that could predict accuracy, including the method of reproduction, musical expertise, reference tempo, and control variables, namely mood, alertness, familiarity and popularity of the songs, and accompanying strategies.

Regarding the *reproduction method*, our findings revealed that participants demonstrated higher accuracy when adjusting the speed of the audio file compared with tapping the beat from memory. These findings confirm the results of the aforementioned study by Jakubowski et al. (2016) with a larger sample, indicating that participants achieved greater accuracy in remembering musical tempo when they adjusted the tempo of an audio file, as opposed to relying on tapping or adjusting a metronome tempo. Similarly, but related to mental representations of pitches, Janata (2012) found that participants showed higher accuracy when they had sensory support, i.e., when they could actually hear the pitches rather than relying only on their imagination. Additionally, a memory study on non-music-related duration recall by Bueti and Walsh (2010) revealed that individuals remembered auditory and visual presentations of time units more accurately when they were required to recognize them rather than reproduce them motorically. Therefore, the observed difference in accuracy between tapping and adjusting the audio file may be attributed to the amount of musical information involved in each method. While tapping engages the motor system and thereby improves tempo production accuracy due to sensorimotor integration compared with merely imagining (Manning & Schutz, 2013), hearing and adjusting the audio file additionally allows participants to register musical details besides the beats, such as rhythms, melodic sequences, and sound changes. Our findings thus suggest that in the realm of musical tempo memory providing people with more musical information may be more beneficial than a task initiating motor engagement. Just as individuals are able to infer a whole song from its first milliseconds (e.g., Schellenberg et al., 1999), the tempo adjusting method may activate the whole neural memory network associated with a song and thus improve tempo recall.

Another central hypothesis was that *musical expertise* would be associated with higher accuracy of musical tempo memory. While some previous studies have shown an advantage for musically trained individuals in specific contexts, such as tempo identification tasks (Foster et al., 2021; Gratton et al., 2016) or tapping tasks (Jakubowski et al., 2016), others could not detect any effect (e.g., Fine & Bull, 2009). In our study, musical expertise significantly predicted accuracy, even if all other predictors and control variables were included in the same model. The finding that musical expertise was associated with higher accuracy in both reproduction methods is noteworthy considering that tapping tasks typically involve two sources of variability: tempo memory and motor execution (Repp, 2005). One might speculate that the advantage observed especially in tapping tasks for musically trained individuals, as indicated by Jakubowski et al. (2016), could be attributed to nonmusicians facing challenges in motor execution (Sowiński & Dalla Bella, 2013), despite having a good tempo memory. However, our results contradict this theory, as higher expertise was associated with greater accuracy in adjusting the tempo of the audio file as well. The stronger effect of expertise in the tapping tasks, thus the interaction between method and expertise, may be attributed to auditory-motor synchronization, but also to heightened mental imagination abilities of musicians (Aleman et al., 2000), as more imagery is needed when no sensory support is available.

The observed effect of musical expertise could further be attributed to factors that influence different phases of the memory process—namely, encoding, storage, and retrieval (McDermott & Roediger, 2018). Regarding encoding, previous research has consistently shown that musicians possess enhanced auditory processing abilities, including discriminating tempo, rhythm, frequency, and pitch (Rammsayer & Altenmüller, 2006; Tervaniemi et al., 2005). Musical training further facilitates the perception of periodicities in musical rhythms and thus beat perception (Spiech et al., 2023), the recognition of crucial tempo-related elements in music, and the detection of small time changes in regular auditory sequences (Jones & Yee, 1997; Nave-Blodgett et al., 2021; Sheldon, 1994). Additionally, musicians’ practice routines involve exploring various tempi, experimenting with rhythmic variations, and adapting to tempo changes in ensemble settings (Allingham & Wöllner, 2022; Collier & Collier, 1994; Keller & Appel, 2010), which may contribute to a heightened awareness of different tempo gradations and subsequently to a more precise storage of distinct tempos.

In terms of storage and recall, according to previous results, musicians exhibit higher mental imagery abilities, particularly in the auditory domain (Aleman et al., 2000) and demonstrate superior reproduction of other musical features, such as pitches, chords, loudness levels, and rhythms (Bishop et al., 2013; Janata & Paroo, 2006; Pallesen et al., 2010; Schaal et al., 2015). The results are also consistent with research on time perception: Musically trained individuals, both experts and non-experts, have shown improved accuracy in estimating the duration of auditory stimuli, and there is a correlation between duration reproduction accuracy and musical training (Plastira & Avraamides, 2021; Rammsayer & Altenmüller, 2006). This suggests a potential progressive refinement of specific skills through musical training, leading to an overall improvement in duration reproduction accuracy. Furthermore, musicians were found to possess memory advantages not only in musical memory tasks but also in general short and long-term memory, particularly regarding auditory stimuli (Talamini et al., 2017). The findings in the present study expand the knowledge on musical expertise and cognition with a more nuanced perspective through dissolving the nonmusician versus musician dichotomy. Our results point to music engagement as a continuous resource for facilitating cognitive processes and tempo memory.

Regarding *reference tempo*, our findings revealed a quadratic effect of tempo on accuracy, with the highest accuracy levels around 120 bpm, and lower accuracies at both faster and slower tempos. In the context of tapping, this association could be attributed to individuals’ spontaneous motor tempo, which is typically around 120 bpm (Hammerschmidt et al., 2021; Moelants, 2002). Research suggests that even when individuals are required to synchronize with faster or slower tempos in motor tasks, they tend to revert to their spontaneous motor tempo over time (McAuley et al., 2006). Similarly, in the case of adjusting, the quadratic effect of tempo could be linked to preferred perceived musical tempos, which typically fall within the range of 120 to 130 bpm (McAuley et al., 2006; McKinney & Moelants, 2006; van Noorden & Moelants, 1999). Furthermore, the average tempo found in dance music is around 120 bpm (Moelants, 2002), which suggests that tempos around this range may be deeply anchored in both auditory and motor memory, facilitating their accurate reproduction. The strong correlation between spontaneous tapping rate and preferred perceived tempos (Michaelis et al., 2014) supports the consistency of our findings in both reproduction methods. Our study thus not only replicates the findings of Jakubowski et al. (2016), who observed improved accuracy of tempo reproduction between 50 and 120 bpm, but also introduces a novel finding by demonstrating a quadratic effect of reference tempo on the accuracy of musical tempo memory.

In addition to our main predictors, we examined several *control variables,* some of which were related to accuracy. We found that women and older participants tended to perform worse, possibly due to factors such as lower stability in tapping tempo among women in this study and poorer general memory performance associated with age (Rhodes et al., 2019). Previous research has further indicated gender and age differences in time estimation (Espinosa-Fernández et al., 2003), which could contribute to less accurate storage of tempo information. Mood and alertness did not impact accuracy, contrary to previous findings (Droit-Volet, 2013; Jakubowski, Halpern et al., 2015b), but most of these studies focused on the relationship between arousal and mood and preferred tempos rather than accuracy of tempo reproduction. Familiarity with the songs had a positive effect, corresponding to literature on repetition learning that repeated exposure improves memory representations (Chen et al., 2008). Additionally, when participants chose faster tapping tempos, they exhibited greater accuracy in tempo reproduction. This aligns with Weber’s law (Madison, 2001), suggesting that time interval perception and production variability increases with longer intervals.

Regarding implications of the present findings, tempo and frequency information play an important role not only in music-related contexts but also in everyday situations, encompassing the prediction and adaptation to rhythmic patterns in traffic signals, maintaining a consistent tempo during physical activities, and adjusting speech tempo to facilitate effective communication with others. Therefore, understanding the factors that influence tempo memory, as explored in our study, can provide valuable insights into how individuals perceive and remember temporal information in different contexts. The investigation of musical tempo memory further provides potential for examining the effects of ageing, brain dysfunction, and neurodegenerative diseases on cognitive functioning. For instance, patients diagnosed with Parkinson’s disease may experience challenges in perceiving and recreating tempos because of impaired coordinated rhythmic locomotion (Nombela et al., 2013). Developing diagnostic tools based on musical tempo may thus facilitate the assessment of executive and cognitive functioning. Practical applications of the study’s findings can be found in the optimization of music education (e.g., tempo memory tests for assessing musical skills, see also Georgi et al., 2023), tempo-based interventions (e.g., rhythmic auditory stimulation) or sports psychology (e.g., using different tempos in music to enhance athletic performance and motivation).

## Limitations

The results of the study should be considered in light of some limitations. Although our sample consisted of a large number of nonmusicians, amateur musicians, and professional musicians spanning different age groups, it was predominantly young and highly educated from Western countries, which may limit the generalizability of the findings to the broader population.

Furthermore, the order of the two reproduction methods introduces a potential limitation. Participants first tapped the tempo and then adjusted the speed of the audio file based on their initial tapping tempo. This sequential order may have influenced the observed accuracy differences between the two methods due to an anchoring effect, where the initial tapped tempo acts as a reference point for subsequent adjustments. Therefore, it remains uncertain if the accuracy of tempo adjustment would have differed with random starting tempos of the audio files. Future research should thus consider the randomization of different reproduction methods for eliminating possible confounding effects.

In addition, the selection of songs focused on popular pop and rock songs known to the target population with simple 4/4 time signatures. While the selected genre facilitated the availability of original versions with fixed tempos and clear metrical levels of perceived tempo (McKinney & Moelants, 2006), it restricts the generalizability of the results to other genres or more complex time signatures. It is worth noting that the contemporary trend of accelerating songs on platforms like TikTok (e.g., Mullen, 2023) were not accounted for, which could lead to uncontrolled variations in known song tempos. Replicating the findings with previously unknown musical snippets would therefore be necessary to address the limitation.

Lastly, although the study included several factors that could influence tempo memory, such as musical expertise, mood, alertness, accompanying strategies, and familiarity and liking of the songs, there may be other relevant predictors that were not considered. For instance, music aptitude, reflecting innate talent rather than musical training, or physical factors like resting heart rate (Cellini et al., 2015) could be valuable additions in future studies.

## Conclusion

In summary, our study provides insights into the factors influencing the accuracy of musical tempo memory. We observed overall high precision in musical tempo memory, with participants achieving greater accuracy when adjusting the audio file compared with tapping. This highlights the importance of sensory integration in tempo perception. Moreover, higher musical expertise was associated with increased accuracy in both methods of tempo production, with a stronger effect on tapping, indicating a specific advantage for musically trained individuals. Notably, we found a quadratic effect for the reference tempo, with the greatest precision in reproducing tempos around 120 bpm. Additionally, gender, age, familiarity with the pieces, and accompaniment strategies were found to influence accuracy. Our findings therefore also contribute to an enhanced understanding of general (tempo) memory and cognitive processes. Further research is needed to identify possible additional predictors and to determine whether the advantage observed in musicians is specific to music or also applies to non-musical time or duration information.

### Supplementary Information

Below is the link to the electronic supplementary material.

Supplementary file1 (DOCX 573 KB)

## Footnotes

1 We chose 150% and 75% because of the logarithmic nature of the bpm scale, which means double and half tempo would be correct as well, just tapped in another metre.

2 In evaluating the effect of the reproduction method, we first examined the achieved score distributions. This served in particular to identify a potential ceiling effect, commonly defined as occurring when tasks are too easy such that a large number of participants score at or near the maximum (e.g., Uttl, 2005). Supplementary Figs. S3 and S4 provide a visual representation of the score distributions across all rated songs. Notably, participants demonstrated superior performance with tempo adjusting compared to tempo tapping. However, as visualized in Fig. S3, there is considerable variability in the data and very few individuals reached the maximum score. Figure S4 illustrates the percentage of selected songs for which participants scored above 0.95. The data show considerable variability, indicating that participants did not consistently achieve top scores for the majority of songs.

3 In an exploratory analysis, we tried to determine if there are other factors that vary in their influence on accuracy depending on musical expertise. We conducted seven additional multilevel models, each incorporating an interaction term with musical expertise into our original model. The results, presented in Tables S5 and S6 in the Supplementary Material, showed no further significant interaction effects.
