# Supplementary material for: Exploring the accuracy of musical tempo memory: The effects of reproduction method, reference tempo, and musical expertise
Source: Mem Cognit. 2024 Mar 20;52(6):1299–312. doi: 10.3758/s13421-024-01543-6 (PMC11362532; doi:10.3758/s13421-024-01543-6)
Supplement: Supplementary file 1 — Supplementary file1 (DOCX 573 KB) [file 13421_2024_1543_MOESM1_ESM.docx]

# Exploring the Accuracy of Musical Tempo Memory: The Effects of Reproduction Method, Reference Tempo, and Musical Expertise

Supplemental Online Material

**Table S1**

*Differences Between the Three Groups of Non-musicians, Amateur Musicians and Professional Musicians, Determined Using Analyses of Variance (ANOVAs).*

|  | **Non-**  **musicians (n = 105)** | **Amateur musicians**  **(n = 137)** | **Professional Musicians**  **(n = 161)** |  |  |  |
| --- | --- | --- | --- | --- | --- | --- |
| Variable | M (SD) | M (SD) | M (SD) | *F* | *df* | *p* |
| **Age** | 26.36 (9.36) | 27.88 (11.56) | 26.43 (9.97) | 0.91 | 2,400 | .405 |
| **Education**^a^ | 4.03 (1.08) | 4.12 (1.36) | 4.36 (1.14) | 2.74 | 2,400 | .066 |
| **Play an instrument** | 23% | 97% | 100% | 398.92 | 2,400 | **<.001** |
| **Choral experience** | 25% | 71% | 88% | 80.77 | 2,400 | **<.001** |
| **Years of instrumental practice** | 2.49 (6.56) | 15.03 (11.58) | 17.18 (9.96) | 77.41 | 2,400 | **<.001** |
| **Percent of life where an instrument was played** | 8% | 52% | 64% | 239.68 | 2,400 | **<.001** |
| **Weekly practice hours** | 1.53 (3.81) | 8.66 (7.85) | 19.08 (11.71) | 130.00 | 2,400 | **<.001** |
| **Weekly music listening**^b^ | 4.09 (1.28) | 4.31 (1.13) | 4.38 (1.17) | 2.03 | 2,400 | .133 |
| **Familiarity with the songs**^c^ | 3.49 (0.60) | 3.65 (0.59) | 3.65 (0.59) | 2.76 | 2,400 | .065 |
| **Liking of the songs**^c^ | 3.36 (0.60) | 3.55 (0.55) | 3.47 (0.58) | 3.22 | 2,400 | **.041** |

*^a^ 1 = compulsory school, 6 = Master or PhD degree*

*^b^ 1 = never, 5 = each day*

*^c^ 1 = not at all, 5 = very much*

**Table S2**

*List of Songs Used in the Study with Familiarities in the Preliminary Study (N = 69)*

| **Song Name** | **Artist**  **/Band** | **BPM** | **Year** | **Familiarity Age Group 1**  **(n = 23)** | **Familiarity Age Group 1**  **(n = 23)** | **Familiarity Age Group 3**  **(n = 23)** | **Overall familiarity**  **(N = 69)** |
| --- | --- | --- | --- | --- | --- | --- | --- |
| Wake me up When September Ends | Green Day | 53 | 2004 | 47,8% | 78,3% | 65,2% | 63,8% |
| The Lion Sleeps Tonight | The Tokens | 61 | 1961 | 65,2% | 82,6% | 78,3% | 75,4% |
| Don’t Worry, be Happy | Bobby McFerrin | 69 | 1988 | 91,3% | 100,0% | 100,0% | 97,1% |
| Hey Jude | The Beatles | 76 | 1968 | 39,1% | 91,3% | 100,0% | 76,8% |
| Despacito | Luis Fonsi | 89 | 2017 | 82,6% | 95,7% | 43,5% | 74,9% |
| Yesterday | The Beatles | 94 | 1965 | 47,8% | 95,7% | 100,0% | 81,2% |
| Dance Monkey | Tones and I | 98 | 2019 | 91,3% | 73,9% | 34,8% | 66,7% |
| Stayin’ Alive | Bee Gees | 104 | 1977 | 43,5% | 69,6% | 91,3% | 68,1% |
| Eye of the Tiger | Survivor | 109 | 1982 | 69,6% | 91,3% | 73,9% | 78,3% |
| The final Countdown | Europe | 117 | 1986 | 65,2% | 87,0% | 73,9% | 75,4% |
| Poker Face | Lady Gaga | 120 | 2008 | 82,6% | 87,0% | 69,6% | 79,7% |
| Abcdefu | Gayle | 123 | 2022 | 73,9% | 8,7% | 4,3% | 29,0% |
| Pretty Woman | Roy Orbison | 130 | 1964 | 43,5% | 82,6% | 91,3% | 72,5% |
| Mamma Mia | Abba | 136 | 2008 | 91,3% | 95,7% | 91,3% | 92,8% |
| Summer of 69 | Bryan Adams | 140 | 1984 | 39,1% | 87,0% | 78,3% | 68,1% |
| La Bamba | Ritchie Valens | 150 | 2018 | 67,5% | 73,1% | 79,1% | 73,2% |
| Good 4 u | Olivia Rodrigo | 167 | 2021 | 82,6% | 26,1% | 13,0% | 40,6% |
| Take on me | A-Ha | 169 | 1985 | 69,6% | 73,9% | 73,9% | 72,5% |

*Note*. Age Group 1: < 23 years, Age Group 2: 23-41 years, Age Group 3: > 41 years.

**Figure S1**

*Visualisation of the Accuracy Scores for an Example Tempo of 100 bpm. The y-axis Represents the Accuracy Score a Participant receives when Reproducing the bpm of the x-axis. The Vertical Reference Line Indicates the Target Tempo of 100 bpm.*


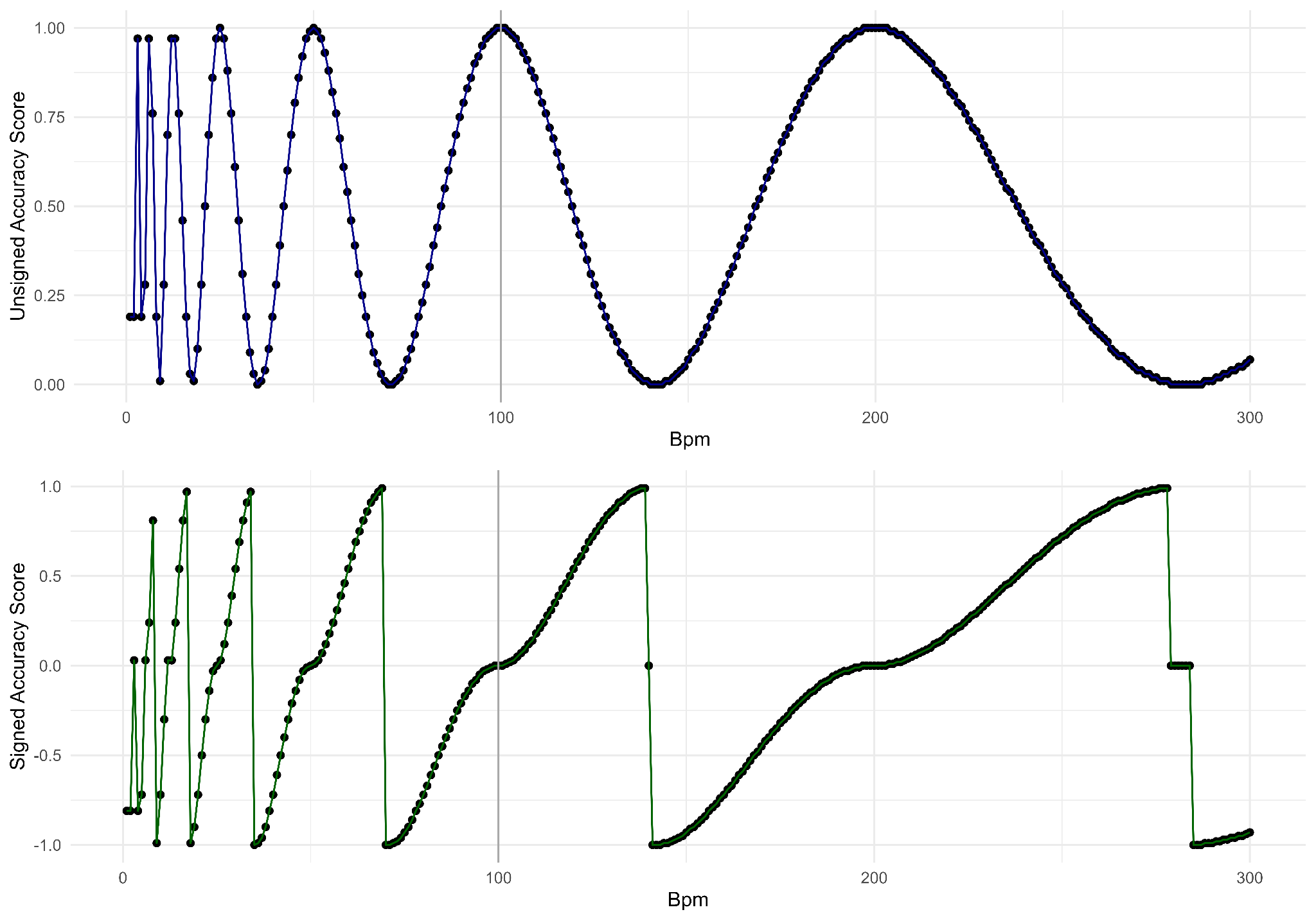


**Figure S2**

*Percentage of Participants who Tapped at the Designated, Half, or Double Metre Across the Different Tempos.*

**
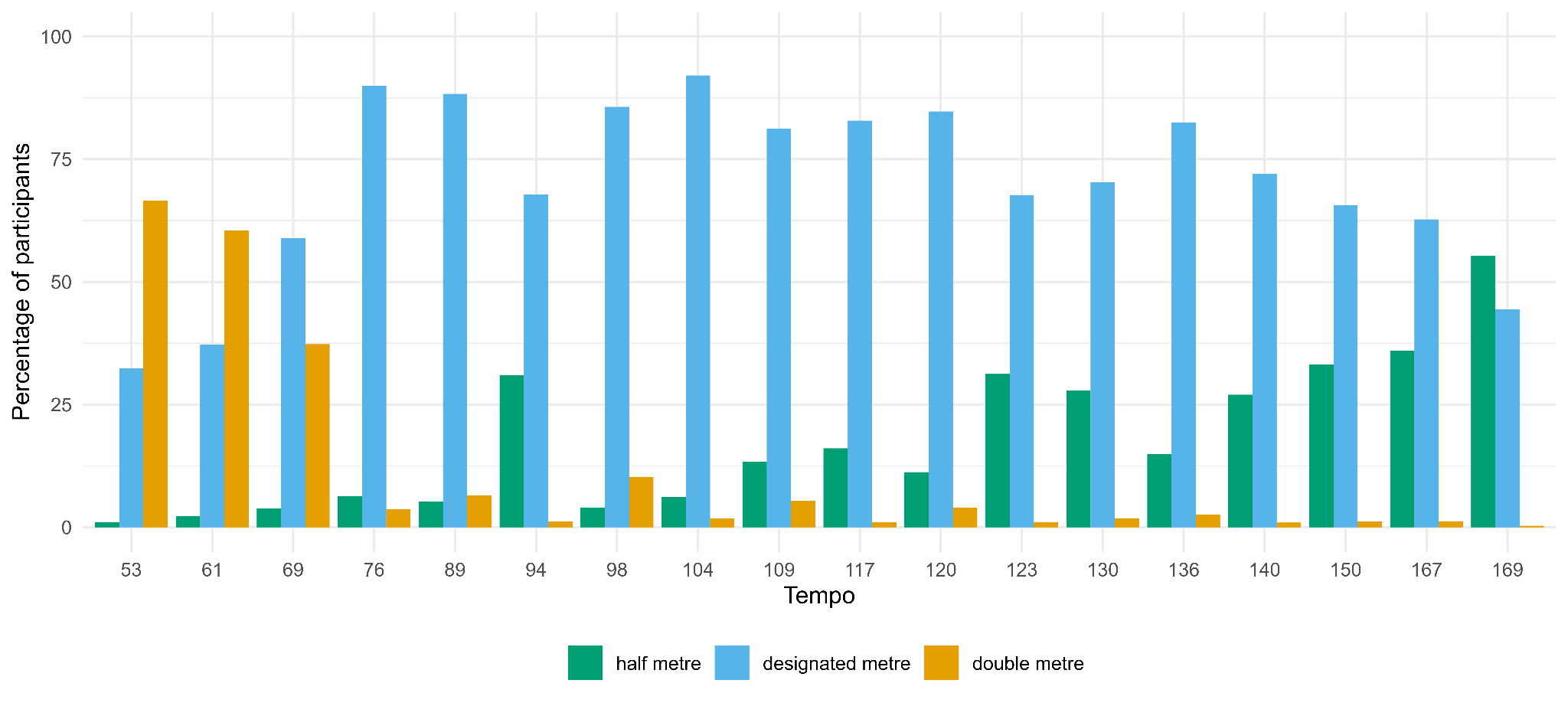
**

**Table S3**

*Comparison of Multilevel Models with Different Random Parameters*

| **Model** | **df** | **AIC** | **BIC** | **logLik** | **Test** | **L.Ratio** | **p-value** |
| --- | --- | --- | --- | --- | --- | --- | --- |
| Random Intercept Model | 3 | -439.92 | -418.05 | 222.96 |  |  |  |
| Model with Random Intercept and Random Slope of Tempo | 5 | -561.24 | -524.80 | 285.62 | 1 vs 2 | 125.32 | <.0001 |

**Table S4**

*Accuracy of Musical Tempo Reproduction, Predicted by a Linear and a Quadratic Effect of Tempo*

| **Predictor** | Est. | CI | *p* | *r* | Est. | CI | *p* | *r* |
| --- | --- | --- | --- | --- | --- | --- | --- | --- |
| (Intercept) | 0.82 | 0.81 – 0.83 | **<.001** |  | 0.81 | 0.80 – 0.82 | **<.001** |  |
| Tempo | 0.01 | 0.01 – 0.02 | **<.001** | .20 | 0.19 | 0.16 – 0.22 | **<.001** | .13 |
| Tempo^2^ |  |  |  |  | -0.18 | -0.21 – -0.15 | **<.001** | .12 |
| **Random effects** | | | | | | | | |
| σ^2^ | 0.05 | | | | 0.05 | | | |
| τ_00_ | 0.01 _id_ | | | | 0.01 _id_ | | | |
| τ_11_ | 0.00 _id.Tempo_ | | | | 0.00 _id.Tempo_ | | | |
| ρ_01_ | 0.13 _id_ | | | | 0.14 _id_ | | | |
| ICC | 0.19 | | | | 0.19 | | | |
| N | 403 _id_ | | | | 403 _id_ | | | |
| Observations | 10812 | | | | 10812 | | | |
| Marginal *R*^2^/  Conditional *R*^2^ | 0.002 / 0.191 | | | | 0.014 / 0.202 | | | |
| *Note.* Effect-size *r* was calculated as √(*t*^2^/(*t*^2^ + *df*)) (Rosenthal & Rosnow, 2008)).  Abbreviations: CI, 95% confidence intervals; Est., unstandardized coefficients; σ^2^ = within-group variance, residual variance; τ_00_ _id_ = between-group variance, random intercept of participant (with random slope of tempo); τ_11_ _id.Tempo_ = variance of the random slope of tempo at participant level; ρ_01_ _id_ = correlation between random intercept of participant and random slope of tempo; ICC = intraclass correlation coefficient. | | | | | | | | |

**Figure S3**

*Distribution of the Average Accuracy Score Over All Songs, Separately for Non-musicians, Amateur Musicians, and Professional Musicians.*


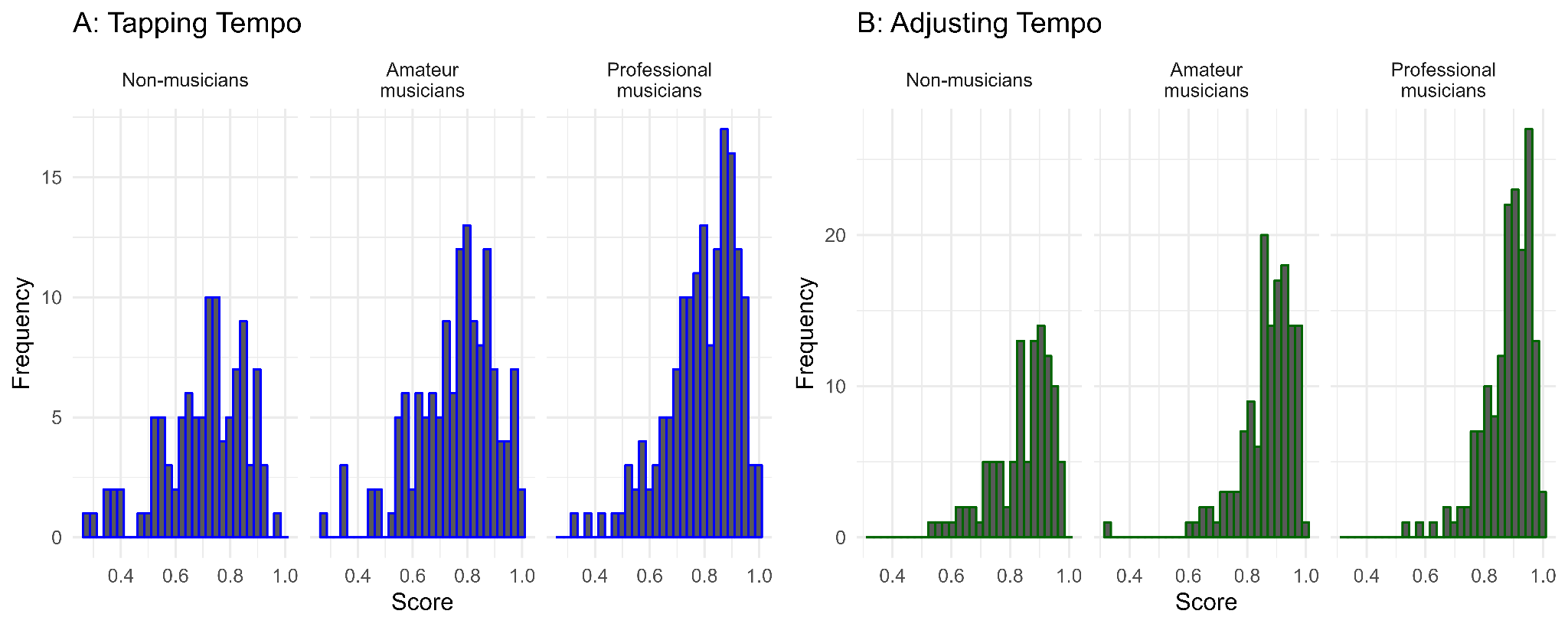


**Figure S4**

*Percentage of Songs on Which Participants Scored Above 0.95, Separately for Non-musicians, Amateur Musicians, and Professional Musicians.*


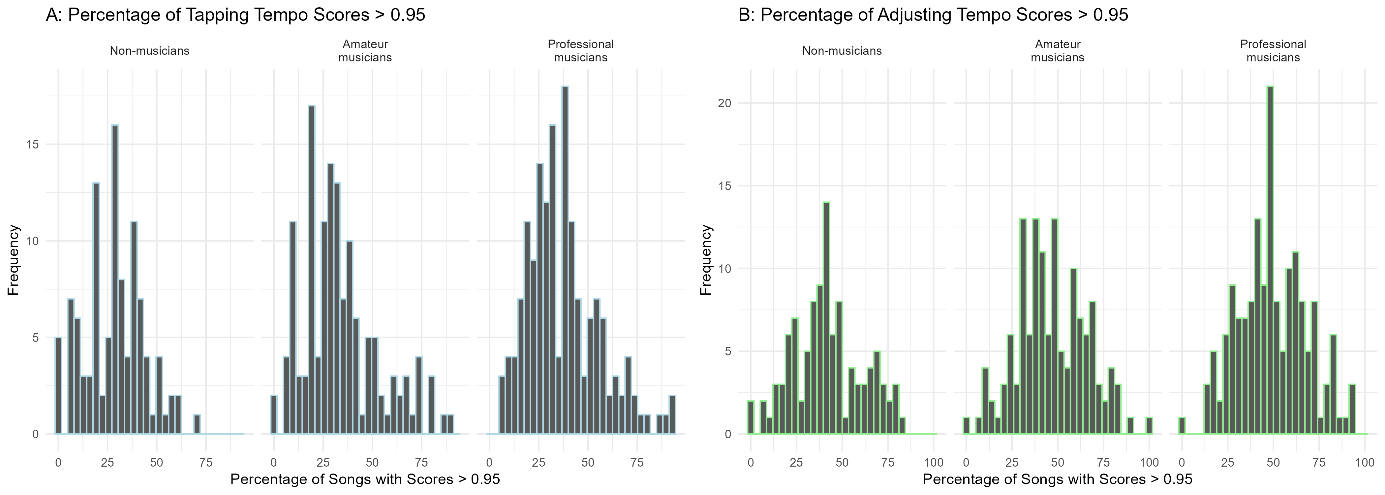


**Exploratory Analysis**

**Table S5**

*Multilevel Models Investigating Interaction Effects Between Musical Expertise and Tempo (Model 1), Positive Affectivity (Model 2), Negative Affectivity (Model 3), and Alertness (Model 4)*

|  | **Model 1** | | **Model 2** | | **Model 3** | | **Model 4** | |
| --- | --- | --- | --- | --- | --- | --- | --- | --- |
| **Predictors** | Est. | p | Est. | p | Est. | p | Est. | p |
| (Intercept) | 0.89 | **<.001** | 0.89 | **<.001** | 0.89 | **<.001** | 0.89 | **<.001** |
| *Tempo* | 0.23 | **<.001** | 0.23 | **<.001** | 0.23 | **<.001** | 0.23 | **<.001** |
| Tempo^2^ | -0.2 | **<.001** | -0.2 | **<.001** | -0.2 | **<.001** | -0.2 | **<.001** |
| Method::Tapping^a^ | -0.11 | **<.001** | -0.11 | **<.001** | -0.11 | **<.001** | -0.11 | **<.001** |
| Musical Expertise | 0.01 | **.048** | 0.01 | .050 | 0.01 | **.039** | 0.01 | .054 |
| Method × Musical Expertise | 0.02 | **<.001** | 0.02 | **<.001** | 0.02 | **<.001** | 0.02 | **<.001** |
| **Control Variables** |  |  |  |  |  |  |  |  |
| Gender::Female^b^ | -0.04 | **<.001** | -0.04 | **<.001** | -0.04 | **<.001** | -0.04 | **<.001** |
| Age | -0.02 | **<.001** | -0.02 | **<.001** | -0.02 | **<.001** | -0.02 | **<.001** |
| Positive Affectivity | 0 | .516 | 0 | .482 | 0 | .651 | 0 | .514 |
| Negative Affectivity | 0 | .316 | 0 | .314 | -0.01 | .277 | 0 | .331 |
| Alertness | -0.01 | .109 | -0.01 | .108 | -0.01 | .185 | -0.01 | .105 |
| Chosen Metre | 0.03 | **<.001** | 0.03 | **<.001** | 0.03 | **<.001** | 0.03 | **<.001** |
| Tapping Variability | -0.01 | **<.001** | -0.01 | **<.001** | -0.01 | **<.001** | -0.01 | **<.001** |
| Familiarity with Song | 0.02 | **<.001** | 0.02 | **<.001** | 0.02 | **<.001** | 0.02 | **<.001** |
| Liking of Song | 0 | .151 | 0 | .143 | 0 | .161 | 0 | .147 |
| Accompanying strategies | 0.01 | .189 | 0.01 | .194 | 0.01 | .165 | 0.01 | .189 |
| Tempo × Musical Expertise | 0.02 | .269 |  |  |  |  |  |  |
| Tempo^2^ × Musical Expertise | -0.01 | .325 |  |  |  |  |  |  |
| Positive Affectivity × Musical Expertise |  |  | -0.01 | .302 |  |  |  |  |
| Negative Affectivity × Musical Expertise |  |  |  |  | 0.01 | .052 |  |  |
| Alertness × Musical Expertise |  |  |  |  |  |  | 0.00 | .727 |
| **Random Effects** | | | | | | | | |
| σ^2^ | 0.05 | | 0.05 | | 0.05 | | 0.05 | |
| τ_00_ | 0.00 _id_ | | 0.00 _id_ | | 0.00 _id_ | | 0.00 _id_ | |
| τ_11_ | 0.00 _id.Tempo_MC_ | | 0.00 _id.Tempo_MC_ | | 0.00 _id.Tempo_MC_ | | 0.00 _id.Tempo_MC_ | |
| ρ_01_ | 1.00 _id_ | | 0.35 _id_ | | 1.00 _id_ | | 0.20 _id_ | |
| ICC | 0.04 | | 0.04 | | 0.04 | | 0.05 | |
| N | 403 _id_ | | 403 _id_ | | 403 _id_ | | 403 _id_ | |
| Observations | 10812 | | 10812 | | 10812 | | 10812 | |
| Marginal R^2^ / Conditional R^2^ | 0.122 / 0.159 | | 0.122 / 0.159 | | 0.124 / 0.161 | | 0.121 / 0.161 | |
| *Note.* Effect-size *r* was calculated as √(*t*^2^/(*t*^2^ + *df*)) [(Rosnow & Rosenthal, 2008)](https://www.zotero.org/google-docs/?rHFIoP).  CI = 95% confidence intervals; Est. = estimates of unstandardized coefficients;  σ^2^ = within-group variance, residual variance; τ_00_ _id_ = between-group variance, random intercept of participant (with random slope of tempo); τ_11_ _id.Tempo_ = variance of the random slope of tempo at participant level; ρ_01_ _id_ = correlation between random intercept of participant and random slope of tempo; ICC = intraclass correlation coefficient.  ^a^Method::Tapping was coded as 0 = adjusting tempo, 1 = tapping tempo.  ^b^Gender::Female was coded as 0 = male or non-binary, 1 = female.  ^c^Tapping Stability: Lower values indicate higher stability. | | | | | | | | |

**Table S6**

*Multilevel Models Investigating Interaction Effects Between Musical Expertise and Tempo (Model 1), Familiarity with the Songs (Model 5), Liking of the Songs (Model 6), and Accompanying Strategies (Model 7)*

|  | **Model 5** | | **Model 6** | | **Model 7** | |  |  |
| --- | --- | --- | --- | --- | --- | --- | --- | --- |
| **Predictors** | Est. | p | Est. | p | Est. | p |  |  |
| (Intercept) | 0.89 | **<.001** | 0.89 | **<.001** | 0.9 | **<.001** |  |  |
| *Tempo* | 0.23 | **<.001** | 0.23 | **<.001** | 0.23 | **<.001** |  |  |
| Tempo^2^ | -0.2 | **<.001** | -0.2 | **<.001** | -0.2 | **<.001** |  |  |
| Method::Tapping^a^ | -0.11 | **<.001** | -0.11 | **<.001** | -0.11 | **<.001** |  |  |
| Musical Expertise | 0.01 | **.047** | 0.01 | .053 | 0.01 | .133 |  |  |
| Method × Musical Expertise | 0.02 | **<.001** | 0.02 | **<.001** | 0.02 | **<.001** |  |  |
| **Control Variables** |  |  |  |  |  |  |  |  |
| Gender::Female^b^ | -0.04 | **<.001** | -0.04 | **<.001** | -0.04 | **<.001** |  |  |
| Age | -0.02 | **<.001** | -0.02 | **<.001** | -0.02 | **<.001** |  |  |
| Education | 0 | .516 | 0 | .521 | 0 | .507 |  |  |
| Positive Affectivity | 0 | .313 | -0.01 | .301 | -0.01 | .283 |  |  |
| Negative Affectivity | -0.01 | .109 | -0.01 | .114 | -0.01 | .104 |  |  |
| Alertness | 0.03 | <.001 | 0.03 | <.001 | 0.03 | <.001 |  |  |
| Chosen Metre | -0.01 | **<.001** | -0.01 | **<.001** | -0.01 | **<.001** |  |  |
| Tapping Variability | 0.02 | **<.001** | 0.02 | **<.001** | 0.02 | **<.001** |  |  |
| Familiarity with Song | 0 | **.147** | 0 | **.154** | 0 | **.144** |  |  |
| Liking of Song | 0.01 | .19 | 0.01 | .205 | 0.01 | .243 |  |  |
| Accompanying strategies | -0.04 | <.001 | -0.04 | <.001 | -0.04 | <.001 |  |  |
| Familiarity × Musical Expertise | 0.00 | .915 |  |  |  |  |  |  |
| Liking × Musical Expertise |  |  | 0.00 | .064 |  |  |  |  |
| Accompanying strategies ×  Musical Expertise |  |  |  |  | -0.01 | .155 |  |  |
| **Random Effects** | | | | | | |  |  |
| σ^2^ | 0.05 | | 0.05 | | 0.05 | |  |  |
| τ_00_ | 0.01 _id_ | | 0.01 _id_ | | 0.01 _id_ | |  |  |
| τ_11_ | 0.00 _id.Tempo_MC_ | | 0.00 _id.Tempo_MC_ | | 0.00 _id.Tempo_MC_ | |  |  |
| ρ_01_ | 0.08 _id_ | | 0.08 _id_ | | 0.09 _id_ | |  |  |
| ICC | 0.17 | | 0.17 | | 0.17 | |  |  |
| N | 403 _id_ | | 403 _id_ | | 403 _id_ | |  |  |
| Observations | 10812 | | 10812 | | 10812 | |  |  |
| Marginal R^2^ / Conditional R^2^ | 0.112 / 0.264 | | 0.112 / 0.265 | | 0.113 / 0.264 | |  |  |
| *Note.* Effect-size *r* was calculated as √(*t*^2^/(*t*^2^ + *df*)) [(Rosnow & Rosenthal, 2008)](https://www.zotero.org/google-docs/?rHFIoP).  CI = 95% confidence intervals; Est. = estimates of unstandardized coefficients;  σ^2^ = within-group variance, residual variance; τ_00_ _id_ = between-group variance, random intercept of participant (with random slope of tempo); τ_11_ _id.Tempo_ = variance of the random slope of tempo at participant level; ρ_01_ _id_ = correlation between random intercept of participant and random slope of tempo; ICC = intraclass correlation coefficient.  ^a^Method::Tapping was coded as 0 = adjusting tempo, 1 = tapping tempo.  ^b^Gender::Female was coded as 0 = male or non-binary, 1 = female.  ^c^Tapping Stability: Lower values indicate higher stability. | | | | | | | | |

**JavaScript Functions for the Assessment of Musical Tempo**

let prev_millisecs;

let current_millisecs;

let bpm = 0;

let bpm_list = [];

let is_mobile = false;

let bpm_avg = 0;

let current_speed = 0;

let num__required_clicks = 15;

let num_clicks = 0;

let css_tap = "https://www2.uibk.ac.at/downloads/c7201049/bpm_project/css/tap_bpm.css";

function display_delta_bpm(){

let delta_millisecs = current_millisecs - prev_millisecs;

let bpm = Math.round(60000 / delta_millisecs);

bpm_list.push(bpm);

}

function display_final_bpm(){

const n = bpm_list.length

const sum = bpm_list.reduce((a, b) => a + b)

const mean = Math.round(sum / n)

const sd = Math.sqrt(bpm_list.map(x => Math.pow(x - mean, 2)).reduce((a, b) => a + b) / n)

bpm_avg = mean;

$("#bpm").text(bpm_avg);

$(".bpm-feld .answer input").val(bpm_avg);

$(".bpm-list .answer input").val(bpm_list.join(','));

$("#speedcontrolcontainer").parents(".question-container").slideDown(1000);

reset_speed();

$("#audio")[0].play();

}

function tap() {

current_millisecs = new Date().getTime();

num_clicks = num_clicks + 1;

$("#num_clicks").text(num_clicks);

if (num_clicks > 2)

display_delta_bpm();

if (num_clicks == num__required_clicks)

display_final_bpm();

prev_millisecs = current_millisecs;

}

function reset_bpm() {

prev_millisecs = 0;

current_millisecs = 0;

bpm = 0;

num_clicks = 0;

bpm_list = [];

$("#num_clicks").text("0");

$("#bpm").text("0");

}

function import_stylesheet(url){

$('head').append(`<link rel="stylesheet" type="text/css" href="${url}">`);

}

function set_speed(speed){

current_speed = speed;

audio.playbackRate = current_speed;

window.localStorage.pbspeed = current_speed;

}

function clean_bpm(target, bpm) {

min = target * 0.75;

max = target * 1.5;

while(bpm > max)

bpm = bpm / 2;

while(bpm < min)

bpm = bpm * 2;

return bpm;

}

function slower(){

set_speed(current_speed - 0.01);

}

function faster(){

set_speed(current_speed + 0.01);

}

reset_speed = function(){

cleaned_bpm = clean_bpm(correct_bpm, bpm_avg);

$(".bpm-feld-cleaned .answer input").val(cleaned_bpm);

current_speed = Math.round(cleaned_bpm / correct_bpm * 100) / 100;

set_speed(current_speed);

}

function set_final_bpm(){

adjusted_bpm = correct_bpm * current_speed;

$(".bpm-adjust .answer input").val(adjusted_bpm);

bpm_score = (Math.round(((Math.cos(Math.log10(adjusted_bpm/correct_bpm)*20.87229)+1)/2)*100))/100;

$(".bpm-score .answer input").val(bpm_score);

$("#movenextbtn, #movesubmitbtn").click();

}

$(document).ready(function () {

$("#speedcontrolcontainer").parents(".question-container").slideUp(1);

import_stylesheet(css_tap);

audio = $('#audio')[0];

if (window.localStorage.pbspeed)

audio.playbackRate = window.localStorage.pbspeed;

$("#movenextbtn").fadeTo(0, 0)

$(".bpm-feld").css("display", "none");

$(".bpm-feld-cleaned").css("display", "none");

$(".bpm-score").css("display", "none");

$(".bpm-list").css("display", "none");

if ($('#some-element').css('display') == 'block')

is_mobile = true;

});
